# Clustering Molecular Subtypes in Breast Cancer, Immunohistochemical Parameters and Risk of Axillary Nodal Involvement

**DOI:** 10.3390/jpm12091404

**Published:** 2022-08-29

**Authors:** Augusto Pereira, Jaime Siegrist, Santiago Lizarraga, Tirso Pérez-Medina

**Affiliations:** 1 Department of Gynecologic Surgery, Puerta de Hierro University Hospital, 28222 Madrid, Spain; 2 Division of Gynecologic Oncology, La Paz University Hospital, 28046 Madrid, Spain; 3 Department of Obstetrics and Gynecology, Gregorio Marañon University General Hospital, 28009 Madrid, Spain

**Keywords:** breast cancer, axillary metastasis, axillary involvement, axillary positive nodes, immunohistochemical, molecular subtypes

## Abstract

(1) Background: To establish similarities in the risk of axillary lymph node metastasis between different groups of women with breast cancer according to immunohistochemical (IHC) parameters. (2) Methods: Data was collected retrospectively, from 2000 to 2013, of 1058 node-positive breast tumours. All patients were divided according to the St Gallen 2013 criteria and IHC features. The proportion of axillary involvement (pN > pN0; pN > pN1mi; pN > pN1) was calculated for each group. Similarities in axillary nodal dissemination were explored by cluster analysis and association between IHC and risk of axillary disease was studied with multivariate analysis. (3) Results: Among clinico-pathological surrogates of intrinsic subtypes, axillary involvement was more frequent in Luminal-B like HER2 negative (45.8%) and less frequent in Luminal-B HER2 positive (33.8%; *p* = 0.044). Axillary macroscopic involvement was more frequent in Luminal-B like HER2 negative (37.9%) and HER2 positive (37.8%) and less frequent in Luminal-B HER2 positive (25.5%) and Luminal-A like (25.6%; *p* = 0.002). Axillary involvement ≥pN2 was significantly less frequent in Luminal-A like (7.4%; *p* < 0.001). Luminal-A with Luminal-B HER2 positive, and triple-negative with Erb-B2 overexpressing tumours were clustered together regarding any axillary involvement, macroscopic disease or ≥pN2. Among the defined subgroups, axillary metastases were more frequent when Ki67 was higher. In a multivariate analysis, Ki67>14% were associated with a risk of axillary metastases (HR: 1.31; 95% CI, 1.51–6.80; *p* < 0.037). (4) Conclusions: there are two lymphatic drainage pathways of the breast according to the expression of hormone receptor-related genes. Positive-ER tumors are associated with lower axillary involvement and negative-ER tumors and Ki67 > 14% with higher nodal involvement.

## 1. Introduction

Applying the molecular classification of breast cancer (BC) to clinical practice is one of the goals of modern senology. The 2013 St. Gallen International BC Consensus Conference defined a clinical-pathological surrogate that has been widely used [1].

Five intrinsic molecular subtypes of BC (Luminal A, Luminal B, HER2-enriched, Basal-like and Claudin-low) have been identified [2]. Recent advances in molecular genetic studies have contributed to a more accurate molecular BC classification [3]. The knowledge of the molecular profile of breast tumors has benefited BC patients in the selection of therapies adjusted to the tumors’ specific characteristics.

However, some molecular subtypes, such as the Luminal subtypes, have been difficult to classify due to significant discrepancies (up to 30%) in the evaluation of the immunohistochemical (IHC) parameters [4]. The impact of the heterogeneity of these intrinsic molecular subtypes of BC remains to be studied and understood. An aspect to be studied is the role of genetic expression of the intrinsic molecular subtypes in the BC axillary spread pattern, and how it might affect the process of clinical decision making.

The aim of this study was to identify similarities between the risk of axillary lymph node (AxLN) metastases and the BC intrinsic molecular subtypes and IHC variables.

## 2. Materials and Methods

### 2.1. Patients’ Population

Data was collected retrospectively from 1043 BC patients who underwent primary surgery with axillary evaluation at Gregorio Marañón University General Hospital, Madrid (Spain) between 2000 and 2013. No patients received neoadjuvant treatment prior to surgery. An Institutional Review Board approval was obtained.

### 2.2. Pathological Assessment

Abstracted data included age, menopausal status, histological tumor type and grade, tumor size (T), nodal status (N) and unilateral or bilateral location. The IHC parameters reviewed were: estrogen receptor (ER) and progesterone receptor (PR), human epidermal growth factor 2 (HER2) overexpression, Ki67 and p53 values. Additional evaluated data was the overall number of nodes removed, and the number of positive nodes resected. The axilla was studied by sentinel node biopsy, axillary lymphadenectomy or both. AxLN metastases were classed as follows: (a) pN+: any axillary involvement; (b) pN1: macroscopic metastases in 1 to 3 AxLN (excluding micrometastasis); (c) ≥pN2: metastases in 4 to 9 AxLN or more. The ratio of axillary involvement was calculated for each group and subgroup.

Patients were divided according to the St. Gallen 2013 surrogate molecular subtypes. For the purposes of this study, a further subdivision was performed, with the following classification: (a) Luminal A-like: ER positive (+) and PR+, HER2 negative (−), Ki67 “low”, and any p53; (b) Luminal B-like (HER2-): ER+, HER2-, Ki67 “high”, and PR “negative or low”; (c) Luminal B-like (HER2+): ER+, HER2+, any Ki67 and any PR; (d) Erb-B2 overexpression: HER2+, ER-, PR-, any Ki67 and any p53; (e) Triple-negative (TN) (ductal): ER-, PR-, HER2-, any Ki67 and any p53.

PR was defined as “positive or high” when the percentage was greater than 20%, whereas PR was defined as “negative or low” if the ratio was equal to or lower than 20%. Additionally, Ki67 was considered as “high” if the percentage was over 14% and as “low” when the ratio was less than 14%. Finally, p53 was defined as “high” when the percentage was higher than 10%, and as “low” when this figure was less than 10%.

### 2.3. Statistical Analysis

The proportion of axillary involvement (pN > pN0; pN > pN1mic; pN > pN1) was calculated for each group and subgroup. The chi-square test was used to estimate the relationship between IHC parameters and the risk of axillary disease; p values less than 0.10 in the univariate analysis were included in the multivariate analysis. The relative risk was calculated.

Logistic regression analysis was used to describe data and to explain the relationship between axillary involvement and IHC variables. In the multivariate analysis, a p value of less than 0.05 was considered significant. The homogeneity of the groups compared in the statistical analysis was confirmed by a subsequent Bonferroni correction test. Groups of fewer than 17 patients were excluded in the statistical analysis. The statistical analysis was performed using the SPSS statistical package.

### 2.4. Cluster Analysis (CA)

The objective is to create groups with similar data and, as different as possible, obtain a classification of multivariate data. The CA consisted of four steps:

#### 2.4.1. Approach to CA

This is a multivariate method used to identify similarities in axillary nodal spread among groups of BC intrinsic molecular subtypes.

#### 2.4.2. Types of Data and Measures of Distance

Data was obtained from the ratio of pN+, pN1 and ≥pN2 in each category and subcategories of the St. Gallen 2013 surrogate molecular subtypes, allowing us to know the magnitude of the risk or the associated protection to the exposure of the prognostic factor studied. The distance between the observations was measured in each of the analyzed predictors. Squared Euclidean distance as a proximity measure and Ward’s minimum variance agglomerative method were used. All of these variables were standardized prior to clustering.

#### 2.4.3. Hierarchical Agglomerative Method

Starting in separate clusters, the most similar clusters were repeatedly combined in order to reduce the number of clusters. This step-wise regression uses dissimilarities or distances between the observations until all patients are included in one cluster. This combination resulted in a minimum increase in the total within-group sum of squares. If a point is reached where clusters that are dissimilar are combined, the within-group sum of squares noticeably increases.

#### 2.4.4. Selecting the Optimum Number of Clusters

The CA can be represented on a diagram known as a “dendrogram”. The dendrogram is a visual graphic representation of clusters in which similar objects have been joined by links whose position in the diagram is determined by the amount of similarities/dissimilarities between the factors. The analysis is exploratory in nature, and the choice of the optimum number of clusters is arbitrary, and depends on the author’s criterion. The horizontal or X-axis of the dendrogram is the observations, and represents dissimilarity between clusters. The vertical or Y-axis is the distance or “height”, and represents similarities between the clusters. In our study, higher or lower values indicated a higher or poorer frequency of axillary metastases. The CA was performed using the R statistical package.

## 3. Results

### 3.1. Patients’ Characteristics

One thousand and fifty-eight breast tumors from 1043 women were reviewed; demographics and clinical characteristics of BC patients are summarized in Table 1. The mean age of the patients was 57.3 years old (range 22–92 years). The mean number of axillary nodes removed was 10.6 (range 1–41), and the mean number of metastatic nodes was four (range 1–37). Furthermore, of the 1058 BC tumors, 247 (23.4%) underwent sentinel node biopsy, 722 (68.2%) axillary lymphadenectomy and 89 (8.4%) both procedures; axillary involvement was detected in 428 (40.5%) of the BC patients. Results are shown in Table 1 and Table 2.

### 3.2. Statistical Analysis

Among clinical-pathological surrogates of intrinsic subtypes: (a) pN+: was more frequent in Luminal B-like HER2- (45.8%) and less frequent in Luminal B-like HER2+ (33.8%, *p* = 0.044), (b) pN1: was more frequent in Luminal B-like HER2- (37.9%) and HER2+ (37.8%) and less frequent in Luminal B-like HER2+ (25.5%) and Luminal A-like (25.6%, *p* = 0.002) and (c) ≥pN2: was significantly less frequent in Luminal A-like (7.4%, *p* < 0.001).

Among the defined subgroups, axillary metastases were more frequent when Ki67 was >14%. In total, 46.8% of the patients with a Luminal B-like subtype had a high Ki67 expression and were PR+, and 46.9% were PR-. Among patients with Erb-B2 overexpression and high Ki67 expression, 45.7% presented high levels of p53, and 47.2% low levels of p53. In total, 44.4% of the TN tumors had a high expression of both Ki67 and p53.

HER2+ tumors and Ki67 > 14% were associated with the risk of developing axillary metastases and also with its severity. Multivariate analysis showed high Ki67 expression to be an independent prognostic factor for axillary nodal involvement (hazard ratio: 1.31; 95% CI, 1.51–6.80; *p* < 0.037).

### 3.3. Cluster Analysis

#### 3.3.1. First CA

**Figure 1:**
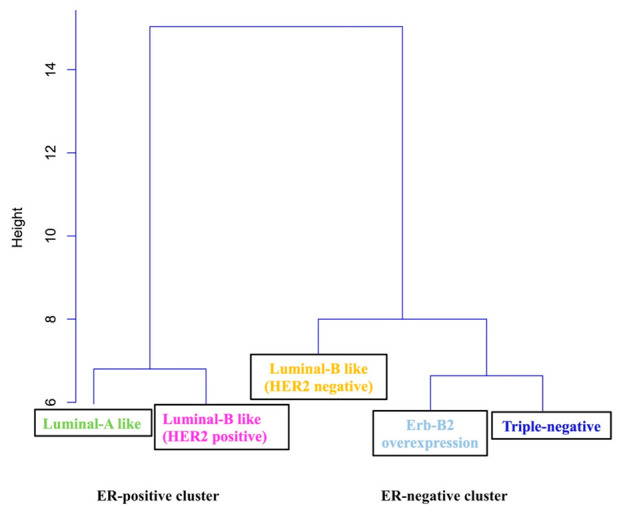
First Cluster Analysis. Abbreviation: HER2: human epidermal growth factor 2.

The agglomeration coefficients generated by CA revealed the possibility between two- and three-cluster solutions. The closest observations to each other were TN and Erb-B2 overexpression, which formed the ER-negative cluster (closest distance to 0); Luminal A-like and Luminal B-like HER2+ formed the ER-positive cluster, both groups being similar due to the minimum distance. The most distinct observation was Luminal B-like HER2-. In a second phase, the distance between the two groups and the observation (Luminal B-like HER2-) were calculated, obtaining a new matrix of distances. The minimum distance was with the first group; therefore, this element was included in the ER-negative cluster. These findings suggest that a two-cluster solution best distinguished the groups in regard to BC axillary node involvement. The resulting two-cluster solution generated relatively well-sized groups related to molecular intrinsic subtypes and gene-expression profiling, and labeled according to their most distinguishing characteristics. In general terms, we consider these results as homogeneous data due to the fact that the majority of the observations are at a distance of less than 2 (in ‘y’ axis). Results are shown in Figure 1.

The ER-positive cluster included those patients with a positive or higher expression of ER-related genes (n = 430; 40.7%). The intrinsic molecular subtypes that composed it showed a lower ratio of axillary nodal involvement in pN+, pN1 and ≥pN2 categories, whereas the ER-negative cluster included negative or lower expression of ER-related genes (n = 626; 59.3%), being characterized by higher ratios of axillary metastasis; results are shown in Table 2.

The results of these first analysis showed that the Luminal B subtype had been separated into both clusters due its HER2 condition, showing a different axillary spread pattern. Due to these results, a secondary CA was performed, including IHC properties of each intrinsic molecular subtype.

#### 3.3.2. Second CA

**Figure 2:**
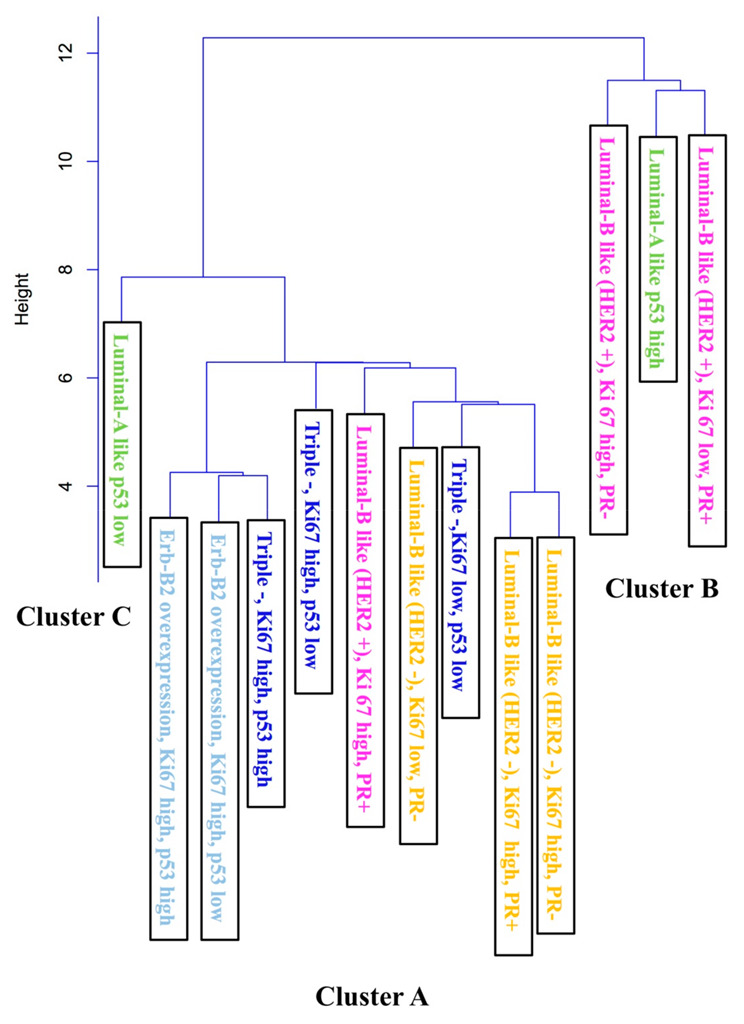
Second Cluster Analysis. Abbreviation: +: positive; -: negative; PR: progesterone receptor; HER2: human epidermal growth factor 2.

The agglomeration coefficients generated by CA revealed the possibility of three-cluster solutions. Results are shown in Figure 2.

Cluster A: It contains nine observations, which are the closest observations to 0 and the most similar between each other. The cluster is composed of two main subgroups:

1. Based on the HER2 negative quality (6 observations): Luminal B, HER2-, high Ki67, PR+ or PR-; Luminal B, HER2-, low Ki67, PR-; TN, with low Ki67 and p53; TN, with high Ki67 and low or high p53.
2. Based on high Ki67 quality (7 observations): TN, high Ki67 and low or high p53; Luminal B, HER2+, high Ki67 and PR+; Luminal B, HER2-, high Ki67, PR+ or PR-; Erb-B2 overexpression, high Ki67 and low or high p53. Results are shown in Figure 2.

Cluster B: Luminal A with low p53 subtype is considered as a different observation, and the next element to join the group, due to the wider distance from the first group.

Cluster C: It is the last group to join and the farthest observation from 0. It comprises Luminal A, high p53; Luminal B, HER2+, low Ki67 and PR-; and Luminal B, HER2+, high Ki67 and PR+.

After the inspection of the clustering tree, the three-cluster solution appears to be appropriate because of the adequate size and correlation with IHC properties and gene-expression profiling: Cluster A includes patients with negative or lower expression of ER-related genes (n = 713; 67.5%). It is the biggest but is considered a homogeneous group because all the observations are at a distance of less than 2 (in ‘y’ axis). Cluster B includes patients with a positive expression of ER and PR-related genes (n=262; 24.8%). Cluster C includes luminal patients with positive expression of ER-related genes (n = 81; 7.7%).

## 4. Discussion

There are many factors that increase the risk of axillary node involvement such as the tumor size and location, histologic grade and lymphatic invasion [5,6]. Furthermore, the presence of perineural and lymphatic vessel invasion, age below 40 years old and extensive intraductal component (>25%) affects the risk of having at least four or more metastatic nodes [7].

The correlation between the intrinsic molecular subtypes and the prognosis, treatment options and risk of recurrence of BC is well-documented [3,8,9,10,11,12,13,14,15,16,17,18,19,20,21]. The role of the intrinsic subtypes in the axillary spread of BC is unclear. Gene-expression profiling has had a considerable impact on our understanding of BC biology. Similarities and differences between gene expressions in molecular subtypes could have an impact on the metastatic BC to regional lymph nodes. Our study could provide some knowledge in this area.

First CA: BC is a heterogeneous group of diseases with different histopathology, genetic profiles and gene expression behavior. Based on molecular phenotypes, our CA suggested that, in BC patients, the existence of two main axillary spread patterns is associated with the expression of hormone receptor (HR)-related genes:

(a) ER-positive cluster (Luminal A and Luminal B HER2+) is associated with lower ratios of nodal involvement (Luminal A: 36.1%; Luminal B HER2+: 33.8%). Luminal subtypes derive from similarities in gene expression of the luminal epithelium of the breast, expressing genes relating to ER. Luminal tumors presented two molecular profiles: Luminal A is characterized by a high expression of ER-related genes, low expression of the HER2 genes and proliferation-related genes [2,21], whereas Luminal B is characterized by low expression of ER-related genes [22], variable expression of HER genes and higher expression of proliferation-related genes [23,24]. Our data suggests Luminal B subtype as the most heterogeneous intrinsic subtype, with a variable gene expression profile and different breast lymphatic pathways (Luminal B HER2-: 45.8%; Luminal B HER2+: 33.8%, *p* = 0.044). On the other hand, the difference between Luminal A and Luminal B HER2- subtypes is the expression of proliferation-related genes.

(b) ER-negative cluster (TN, HER2+, and Luminal B HER2-) is associated with higher ratios of axillary nodal metastases (TN: 40%; HER2+: 42.7%; Luminal B HER2: 45.8%). These differences were statistically significant in all categories of nodal involvement (pN+ *p =* 0.044, pN1 *p =* 0.002, ≥pN2 *p =* 0.001). The HER2-enriched subtype is characterized by an overexpression of the HER2 gene, higher proliferation-related genes and a low expression of the luminal and basal genes [25,26,27,28,29]. The ER-negative genomic profile includes multiple subtypes known as “triple negative”, which are characterized by a lack of HER2 gene amplification, ER and PR expression. The basal-like subtype derives from similarities in gene expression of the basal epithelial cells of normal breast tissue, and is characterized by an overexpression of CK 5/6 and EGFR, and low expression of the luminal and HER2 genes. The claudin-low subtype is characterized by a high expression of mesenchymal markers, immune response genes (lymphocytes and endothelial cell markers) and cancer stem cell-like features, and a low expression of luminal genes, proliferation-related genes, genes involved in tight junctions and epithelial cell-adhesion (claudins 2, 4, 7, occludin and E-cadherin) [30,31,32,33].

Second CA: In order to understand the impact of IHC in the BC spread pattern to the axilla, a secondary CA was performed including all subcategories studied (17). The clustering tree, based on the expression of the HR-related genes, could be modified as follows:

In our statistical analysis, the prognostic value of p53 expression in nodal involvement is likely to be weak, except in Luminal A tumors. The ER-positive cluster can be divided into two subgroups based on p53 evaluation: Luminal A high-p53 together with subtypes of Luminal B (cluster C), and Luminal A low-p53 subtype (cluster B). Usually, the Luminal A subtype is characterized by a lower rate of p53 gene mutation (12%), in contrast with the Luminal B subtype (32%) [3]. The genetic profile of the Luminal A high-p53 subtype resembles the Luminal B subtype.

The ER-negative cluster (cluster A) includes all of the intrinsic subtypes with a negative expression of ER and PR genes, and also some Luminal B tumors, probably due to the lower expression of ER-related genes (around 20% of Luminal B tumors). In addition, all intrinsic subtypes of cluster A showed a higher expression of proliferative genes. Some authors reveal an obvious correlation between Luminal B subtypes and Basal subtypes [34].

The inspection of the clustering tree, and based on high Ki67 expression (7 of 9 subcategories) and HER2- condition (6 of 9 subcategories), suggests a further subdivision of cluster A into two main subgroups. Ki67 is the most common marker of proliferation used by IHC to assess the proliferative rate of BC. Our multivariate analysis showed high Ki67 expression as an independent prognostic factor of BC in axillary involvement (hazard ratio = 1.31). Due to this finding, the distribution of “high Ki67” patients can influence the final result of the CA; in this sense, the Luminal B-like tumors showed a difference close to 10% higher in favor of the Luminal B HER2- patients (especially in pN1 and pN2) (Table 2); thus, this difference may be enough to group these tumors in the ER-negative cluster.

## 5. Conclusions

We can distinguish between two lymphatic drainage pathways of the breast according to the expression of HR-related genes. ER+ breast tumors are correlated with lower axillary involvement, whereas ER- breast tumors are associated with higher nodal involvement. Luminal B should be considered as a “heterogeneous intrinsic subtype” due to its variable axillary nodal behavior. High Ki67 expression could be the key to greater axillary nodal dissemination.

## Figures and Tables

**Table 1:** Characteristics of patients with breast cancer.

Age at Diagnosis	N (%)		Total
≤50 yr	353 (33.4%)	-	
>50 yr	705 (66.6%)	-	1058
Menopausal status	N (%)		Total
Premenopausal	297 (28.1%)	-	
Postmenopausal	756 (71.4%)	-	
Unknown	5 (0.5%)	-	1058
Histopathology	N (%)		Total
Invasive ductal	887 (83.8%)	-	
Invasive lobular	118 (11.2%)	-	
Mixed	53 (5%)	-	1058
Grade	N (%)		Total
I	26 (2.4%)	-	
II	765 (72.3%)	-	
III	263 (24.9%)	-	
Unknown	4 (0.4%)	-	1058
Tumor size	N (%)		Total
T1	695 (65.7%)	-	
T2	323 (30.5%)	-	
T3	22 (2.1%)	-	
Unknown	18 (1.7%)	-	1058
Axillary involvement	No	Yes	Total
Any axillary involvement	628 (59.5%)	428 (40.5%)	1056
≥pN1 (macroscopic involvement)	711 (67.3%)	345 (32.7%)	1056
≥pN2	893 (84.6%)	163 (15.4%)	1056
BC location	Unilateral	Bilateral	Total
	1043 (98.6%)	15 (1.4%)	1058
IHC parameters	Negative	Positive	Total
ER	222 (21%)	836 (79%)	
PR	429 (40.5%)	629 (59.5%)	
HER2	829 (78.4%)	229 (21.6%)	
Ki67	285 (26.9%)	773 (73.1%)	
p53	561 (53%)	497 (47%)	1058
Clinico-pathologic surrogate of the intrinsic subtypes (St. Gallen 2013)	N (%)	Rest (%)	Total
Luminal A	285 (26.9%)	773 (73.1%)	
Luminal B (HER2 negative)	404 (38.2%)	654 (61.8%)	
Luminal B (HER2 positive)	147 (13.9%)	911 (86.1%)	
HER 2 Non luminal	82 (7.8%)	976 (92.2%)	
Triple negative (ductal)	140 (13.2%)	918 (86.8%)	1058

Abbreviation: N: Number of patients; %: Percentage; yr: year; IHC: immunohistochemical; ER: estrogen receptor; PR: progesterone receptor; HER2: human epidermal growth factor 2.

**Table 2:** Correlation between categories, subcategories and axillary involvement.

St. Gallen Consensus Categories and Subcategories	Distribution of BC Patients N (%)	Any axillary Involvement. N (%)	Axillary Macroscopic Involvement. N (%)	Axillary Involvement pN2 or More. N (%)
Luminal-A like	285 (26.9)	103 (36.1)	73 (25.6)	21 (7.4)
p53 low	262 (24.8)	99 (37.8)	71 (27.1)	19 (7.3)
p53 high	17 (1.6)	4 (23.5)	2 (11.8)	2 (11.8)
Luminal-B like (HER2 negative)	404 (38.2)	185 (45.8)	153 (37.9)	68 (16.8)
Ki67 high, PR positive	248 (23.4)	116 (46.8)	98 (39.5)	40 (16.1)
Ki67 low, PR negative	75 (7.1)	31 (41.3)	24 (32.0)	12 (16.0)
Ki67 high, PR negative	81 (7.7)	38 (46.9)	31 (38.3)	16 (19.8)
Luminal-B like (HER2 positive)	147 (13.9)	49 (33.8)	37 (25.5)	20 (13.8)
Ki67 high, PR positive	70 (6.6)	25 (35.7)	21 (30.0)	10 (14.3)
Ki67 low, PR negative	11 (1.0)	5 (45.5)	4 (36.4)	3 (27.3)
Ki67 high, PR negative	40 (3.8)	13 (34.2)	6 (15.8)	4 (10.5)
Ki67 low, PR positive	26 (2.5)	6 (23.1)	6 (23.1)	3 (11.5)
HER2 overexpression	82 (7.8)	35 (42.7)	31 (37.8)	23 (28.0)
Ki67 low, p53 low	6 (0.6)	0 (0.0)	0 (0.0)	0 (0.0)
Ki67 high, p53 low	36 (3.4)	17 (47.2)	15 (41.7)	10 (27.8)
Ki67 high, p53 high	35 (3.3)	16 (45.7)	14 (40.0)	11 (31.4)
Ki67 low, p53 high	3 (0.3)	1 (33.3)	1 (33.3)	1 (33.3)
Triple-negative	140 (13.2)	56 (40.0)	51 (36.4)	31 (22.1)
Ki67 low, p53 low	24 (2.3)	10 (41.7)	9 (37.5)	4 (16.7)
Ki67 high, p53 low	45 (4.3)	15 (33.3)	14 (31.1)	9 (20.0)
Ki67 high, p53 high	63 (6)	28 (44.4)	25 (39.7)	16 (25.4)
Ki67 low, p53 high	6 (0.6)	2 (33.3)	2 (33.3)	2 (33.3)

Abbreviation: N: Number of patients; %: Percentage; BC: Breast cancer; HER2: human epidermal growth factor 2.

## Data Availability

Data presented in this manuscript are available from the corresponding authors on reasonable request.
